# Multiplex Droplet Digital Polymerase Chain Reaction Assay for Rapid Molecular Detection of Pathogens in Patients With Sepsis: Protocol for an Assay Development Study

**DOI:** 10.2196/33746

**Published:** 2021-12-13

**Authors:** Samir Badran, Ming Chen, John E Coia

**Affiliations:** 1 Research Unit in Clinical Microbiology Department of Clinical Diagnostics Hospital South West Jutland, University Hospital of Southern Denmark Esbjerg Denmark; 2 Department of Regional Health Research Faculty of Health Sciences University of Southern Denmark Odense Denmark; 3 Department of Clinical Microbiology Hospital of Southern Jutland, University Hospital of Southern Denmark Aabenraa Denmark

**Keywords:** sepsis, ddPCR, clinical microbiology, molecular diagnostics, infectious diseases

## Abstract

**Background:**

Blood cultures are the cornerstone of diagnosis for detecting the presence of bacteria or fungi in the blood, with an average detection time of 48 hours and failure to detect a pathogen occurring in approximately 50% of patients with sepsis. Rapid diagnosis would facilitate earlier treatment and/or an earlier switch to narrow-spectrum antibiotics.

**Objective:**

The aim of this study is to develop and implement a multiplex droplet digital polymerase chain reaction (ddPCR) assay as a routine diagnostic tool in the detection and identification of pathogens from whole blood and/or blood culture after 3 hours of incubation.

**Methods:**

The study consists of three phases: (1) design of primer-probe pairs for accurate and reliable quantification of the most common sepsis-causing microorganisms using a multiplex reaction, (2) determination of the analytical sensitivity and specificity of the multiplex ddPCR assay, and (3) a clinical study in patients with sepsis using the assay. The QX200 Droplet Digital PCR System will be used for the detection of the following species-specific genes in blood from patients with sepsis: *coa* (staphylocoagulase) in *Staphylococcus aureus*, *cpsA* (capsular polysaccharide) in *Streptococcus pneumoniae*, *uidA* (beta-D-glucuronidase) in *Escherichia coli*, *oprL* (peptidoglycan-associated lipoprotein) in *Pseudomonas aeruginosa*, and the highly conserved regions of the 16S rRNA gene for Gram-positive and Gram-negative bacteria. All data will be analyzed using QuantaSoft Analysis Pro Software.

**Results:**

In phase 1, to determine the optimal annealing temperature for the designed primer-probe pairs, results from a gradient temperature experiment will be collected and the limit of detection (LOD) of the assay will be determined. In phase 2, results for the analytical sensitivity and specificity of the assay will be obtained after an optimization of the extraction and purification method in spiked blood. In phase 3, clinical sensitivity and specificity as compared to the standard blood culture technique will be determined using 301 clinical samples.

**Conclusions:**

Successful design of primer-probe pairs in the first phase and subsequent optimization and determination of the LOD will allow progression to phase 3 to compare the novel method with existing blood culture methods.

**International Registered Report Identifier (IRRID):**

PRR1-10.2196/33746

## Introduction

Sepsis, a dysregulated host response to infection leading to life-threatening organ dysfunction [1], often caused by bloodstream infection (BSI), is a major public health concern worldwide. Sepsis affects more than 48 million people annually, including an estimated 3 million newborns, leading to more than 11 million deaths annually, mainly in a hospital setting [2,3]. This makes it one of the leading causes of death worldwide [4]. In Denmark, the incidence rate is estimated to be 56,145 cases per year with a mortality of 8085, potentially accounting for 15% of all deaths [5,6].

Most sepsis survivors experience additional morbidities, resulting in reduced physical and mental quality of life after diagnosis [7,8]. Up to 32% of patients with sepsis have a rehospitalization episode within 30 days and 60% are readmitted at least once within one year [8]. According to a Danish study by Perner et al [9], more than 50% of sepsis survivors die in the first year following diagnosis. The significant burden of morbidity and mortality from sepsis has a profound impact on patients and their families, and it is a substantial economic burden on health care systems and society [10].

Sepsis is a profound inflammatory response to infections caused by bacterial, viral, fungal, or parasitic pathogens [11]. One of the primary reasons for the high morbidity and mortality rate of sepsis is delay in diagnosis and initiation of antimicrobial therapy—every hour of delay in appropriate antimicrobial treatment increases mortality by 7.6% [12,13]. As many as 80% of sepsis deaths could be prevented with rapid diagnosis and treatment [14]. During BSI, the bacterial load is estimated to be 1-10 CFU/mL [15] or 10^3^ to 10^4^ copies of bacterial DNA/mL [16]. Blood cultures are the cornerstone of microbiological diagnosis of sepsis. Key limitations are low sensitivity and long detection time (24-72 hours), with failure to detect a pathogen occurring in approximately 50% of patients with sepsis [15,17,18]. Gupta et al [19] have shown that sepsis-associated mortality was significantly higher in patients with a negative blood culture (34.6%) compared to patients with a positive blood culture (22.7%). Infection with fastidious microorganisms, antimicrobial treatment prior to blood collection, and low bacterial load all contribute to the occurrence of false-negative blood culture [15,20].

Multiplex real-time quantitative polymerase chain reaction (qPCR) has been increasingly employed in combination with positive blood culture to increase diagnostic sensitivity in patients with sepsis [21]. Multiplex qPCR also facilitates more rapid diagnosis [21-23], as demonstrated for the commercially available Septifast (Roche Diagnostics) [24] and FilmArray Blood Culture ID Panel (BCID; BioFire Diagnostics) [25]. The use of multiplex qPCR demonstrated high concordance with the blood culture technique, with up to 100% specificity and a limit of detection (LOD) ranging from 1 to 10 CFU/reaction [22,24]. It has been reported in some studies that multiplex qPCR detected the presence of a pathogen in 10%-40% of cases that were negative by conventional blood culture [26-28]. However, other studies have shown a reduced sensitivity, ranging from 28%-66%, in comparison with conventional blood cultures [28-30]. It is apparent that there is still a need for techniques to improve the diagnostic yield and reduce the time to diagnosis from blood culture specimens. The combination of nanoliter-sized droplet technology paired with digital polymerase chain reaction (PCR), known as droplet digital PCR (ddPCR), is a novel diagnostic tool that partitions the reaction into up to 20,000 droplets before amplification [31]. This method provides absolute quantification of target sequences and has demonstrated greater sensitivity, reproducibility, precision, and accuracy compared to qPCR [32-34]. For instance, the sensitivity of ddPCR was 6.4 copies/20 μL reaction for plasmid DNA and 5 CFUs/20 μL reaction for bacterial cells as compared to 12 copies/20 μL reaction and 36 CFUs/20 μL reaction using qPCR, respectively [35]. Furthermore, a study by Dong-Ku et al [36] demonstrated that, by using droplet digital detection technology, they were able to detect bacteria at the single-cell level in unprocessed diluted blood.

Recently, ddPCR has been investigated as a novel technique for the detection of pathogens in BSI. Wouters et al [37] demonstrated an overall sensitivity and specificity of 80% and 87%, respectively. Furthermore, they were able to detect *Escherichia coli* at a 10- to 100-fold lower concentration when compared to qPCR and with a detection limit of approximately 1-2 bacteria. Zhang et al [38] demonstrated similar results, with a detection rate up to 80%-90% of *Staphylococcus aureus* and *E coli* in blood when using ddPCR; both studies had promising results.

In this study, we will investigate ddPCR as a novel technique for sepsis diagnosis from whole blood and blood culture after 3 and 72 hours of incubation. To the best of our knowledge, this would be the first study that involves developing and implementing a multiplex ddPCR assay as a routine diagnostic tool for early detection of the most common sepsis-causing pathogens (ie, *S aureus, Streptococcus pneumoniae*, *E coli*, and *Pseudomonas aeruginosa*) in patients with sepsis. We believe that the technique will subsequently support clinicians to initiate early and rational antimicrobial treatment by reducing processing time and increasing the detection rate in blood cultures. Improved microbiological diagnosis of sepsis will not only help improve outcomes for sepsis, but also will contribute to improved antimicrobial stewardship and rational antibiotic prescribing.

## Methods

**Figure 1:**
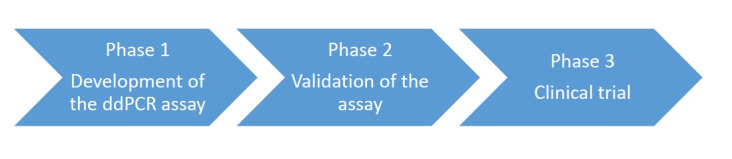
Flowchart showing the three phases of the study, consisting of the development of the assay in phase 1, validation of the assay in phase 2, and a clinical trial using the assay in phase 3. ddPCR: droplet digital polymerase chain reaction.

This study will be conducted in three phases (for flowchart, see Figure 1).

### Phase 1: Establishment of the Multiplex ddPCR Assay for the Detection of Quantitative Bacterial Genomic DNA and Determination of the LOD

In this phase, we will develop a novel multiplex ddPCR assay for accurate and reliable quantification of the genomic DNA of the most common sepsis-causing pathogens in our hospital region (ie, *S aureus, S pneumoniae*, *E coli*, and *P aeruginosa*) from cultured blood. This phase will be conducted in collaboration with the Department of Biochemistry, Hospital of Lillebaelt, Vejle, Denmark, since our laboratory facility does not currently have the QX200 Droplet Digital PCR System (Bio-Rad). This phase will include the following steps:

1. Design of primers and probes for the amplification of species-specific genes—that is, *coa* (staphylocoagulase) in *S aureus, cpsA* (capsular polysaccharide) in *S pneumoniae, uidA* (beta-D-glucuronidase) in *E coli, oprL* (peptidoglycan-associated lipoprotein) in *P aeruginosa*, and the highly conserved regions of the 16S rRNA gene for Gram-positive and Gram-negative bacteria.
2. Determination of the optimal thermocycling conditions for all primer pairs by varying the annealing temperature, extension time, and number of cycles.
3. Determination of the LOD of the assay by using a series of 10-fold serial dilutions of quantitative genomic DNA from a well-characterized stock (10^5^ copies/µL). The serial dilutions of the stock will also be aliquoted and used as positive controls in subsequent analyses.
4. Determination of the rate at which false positives occur per run by analyzing a whole 96-well plate containing only nontemplate controls (ie, saline and uninfected cultured blood samples). Based on the evidence from this experiment, criteria for samples considered to be positive will be determined.

In this phase, primer-probe pairs for the targets of *coa*, *cpsA*, *uidA*, and *oprL* were designed using AlleleID (version 7.85; PREMIER Biosoft). The following ATCC strains were used for preliminary validation and testing of the designed primer-probe pairs: ATCC 29213 (*S aureus*), 49619 (*S pneumoniae*), 25922 (*E coli*), and 27853 (*P aeruginosa*).

### Phase 2: Determination of the Analytical Sensitivity and Specificity of the Multiplex ddPCR Assay Compared to the Blood Culture Technique and BCID Assay in Spiked Blood

This phase will be divided into two parts.

#### Part A

In order to determine the analytical sensitivity and specificity of the multiplex ddPCR assay in cultured clinical samples, we will establish and optimize a procedure for the extraction and purification of bacterial genomic DNA from blood cultures in spiked blood. To confirm the adequacy of the purification procedure, the spiked blood will be compared to saline samples of the chosen bacteria of 1, 5, 10, and 10^2^ CFU/mL from 5 healthy/noninfected donors. Certified reference materials for the spiking of blood samples will be used.

#### Part B

To validate the multiplex ddPCR assay, the analytical sensitivity and specificity will be compared to the blood culture technique and the BCID assay. For this study, a total of 50 blood samples (two BD BACTEC bottles corresponding to 20 mL and 1 mL of whole blood) from healthy/noninfected donors will be used. A total of 48 samples will be spiked with *S aureus, S pneumoniae*, *E coli*, and *P aeruginosa* (12 samples per bacteria) in triplicates of each concentration (ie, 1, 5, 10, and 10^2^ CFU/mL). Two samples will be cultured with saline as nontemplate controls. All samples will be analyzed in duplicate by the multiplex ddPCR and BCID assay in parallel with blood cultures.

### Phase 3: Multiplex ddPCR for Rapid Identification of Pathogens in Cultured Blood and Comparison of the Rate of Detection of Pathogens by Multiplex ddPCR With Conventional Blood Culture in Patients With Sepsis

#### Overview

This phase will investigate the use of the developed multiplex ddPCR assay for clinical samples from 301 patients with suspected sepsis. Patients will be selected for inclusion based on the following criteria: aged ≥18 years, lactate ≥2 mmol/L, and meet at least two of the following criteria, based on scores on the quick Sepsis-related Organ Failure Assessment (qSOFA): hypotension (systolic blood pressure ≤100 mm Hg), respiratory rate ≥22 breaths/minute, or altered mental status [1]. Patients will be excluded if they have no clinical or laboratory signs of sepsis.

**Figure 2:**
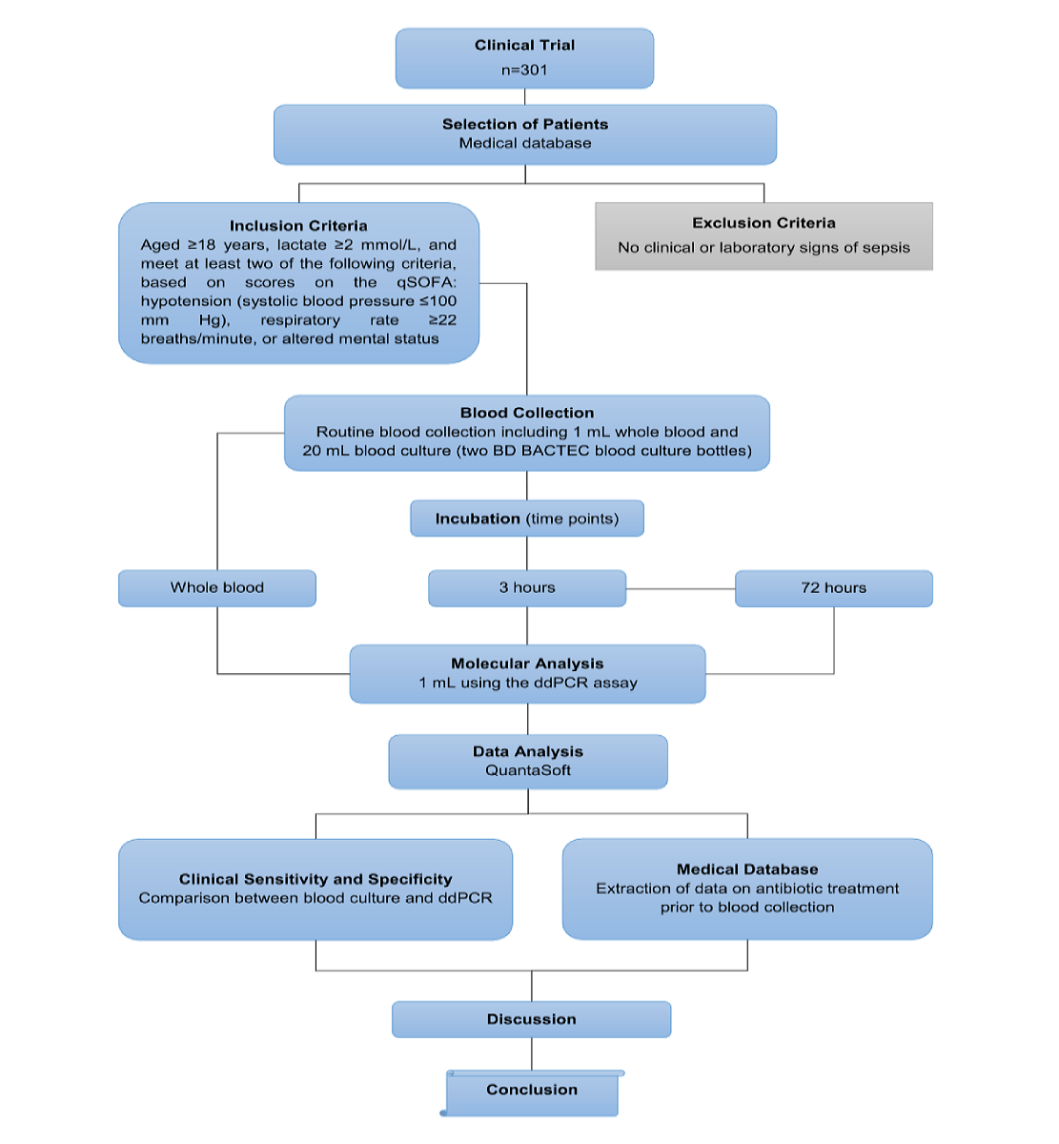
Flowchart showing the study design of the clinical trial in phase 3, including patient selection, molecular detection divided into two parts (whole blood and incubation of blood cultures), and data analysis. ddPCR: droplet digital polymerase chain reaction; qSOFA: quick Sepsis-related Organ Failure Assessment.

The experiment will be blinded, and the results of the ddPCR assay will not be available to the clinical teams before the end of the study. This phase will be divided into two parts (for flowchart, see Figure 2).

#### Part A

The multiplex ddPCR assay will be performed directly on 1 mL of whole blood, and again after 3 hours of incubation in BD BACTEC blood culture bottles. The results of these tests will be compared with those obtained after 72 hours of incubation in conventional blood cultures to compare the rate of detection in clinical samples.

#### Part B

For Part B, 1 mL from conventional blood cultures that remained negative after day 3 will be analyzed retrospectively by ddPCR to confirm negative results [39]. Based on previous studies [26-28] showing that 10%-40% of negative blood cultures were found to be positive using a multiplex qPCR assay, we anticipate that at least 10% of those bottles that are negative by conventional culture will be positive using the multiplex ddPCR assay [32-35]. Data on antibiotic treatment and routine laboratory analyses will be extracted from clinical records and evaluated after collection and analysis of all blood samples. Data from the ddPCR assay will be analyzed using QuantaSoft Analysis Pro Software (Bio-Rad).

The sample size was calculated using Statulator, an online tool, with a power of 80% and a significance level of 5%. The calculation is based on the assumption that 15% and 20% of the pairs are positive by blood culture and ddPCR, respectively, and that the correlation between paired observations is 70%.

### Patient and Public Involvement

Patients and the public were not involved in the design or conduct of the study in any way, since the clinical trial will not influence patient management decisions. Therefore, the results will not be disseminated to the study participants.

### Timeline

The outlined study will be conducted as a regional study within the Region of Southern Denmark. For this study, six months will be allocated to phase 1, five months to phase 2, and 16 months to phase 3. Finally, 14 months in total will be allocated for writing articles in parallel with the experiments. The study started in February 2021.

### Ethics and Dissemination

Blood samples from healthy/noninfected donors will be anonymized and only used for spiking and nontemplate controls. All samples in phase 3 will be collected as part of routine diagnosis and management of patients, and the ddPCR results will not influence patient management decisions. Blood samples from patients with suspected sepsis will be pseudonymized and only used for method comparison. The Regional Committees on Health Research Ethics for Southern Denmark have notified for permission to conduct the study. Since the clinical study in phase 3 will not influence patient management decisions, no approval is required according to the Regional Committees on Health Research Ethics for Southern Denmark.

The results and findings from phases 1, 2, and 3 are expected to be published in peer-reviewed journals, preferably open access. National and at least two international conferences will be attended to present results and liaise with the scientific community. Science channels and the news will also be used to disseminate results.

## Results

### Phase 1: Establishment of the Multiplex ddPCR Assay for the Detection of Quantitative Bacterial Genomic DNA and Determination of the LOD

In phase 1, results from a gradient temperature experiment will be collected to determine the optimal annealing temperature for the designed primer-probe pairs. In addition, the results for the LOD of the multiplex assay will be obtained.

### Phase 2: Determination of the Analytical Sensitivity and Specificity of the Multiplex ddPCR Assay Compared to the Blood Culture Technique and BCID Assay in Spiked Blood

In phase 2, results of the optimized extraction and purification method will be presented and the analytical sensitivity and specificity of the multiplex assay will be obtained using spiked blood samples.

### Phase 3: Multiplex ddPCR for Rapid Identification of Pathogens in Cultured Blood and Comparison of the Rate of Detection of Pathogens by Multiplex ddPCR With Conventional Blood Culture in Patients With Sepsis

In phase 3, the clinical sensitivity and specificity of the multiplex ddPCR assay will be obtained and compared with the blood culture technique using 301 clinical samples.

This study is expected to conclude in February 2024.

## Discussion

Successful design of primer-probe pairs for a multiplex reaction in the first phase—and subsequent optimization and determination of the LOD—will allow progression to phase 2 to determine the analytical sensitivity and specificity of the assay, which will allow progression to phase three to compare the method with existing blood culture methods.

## Appendix

Multimedia Appendix 1 Peer-reviewer report from the University of Southern Denmark, Graduate School - Faculty of Health Sciences.

## Abbreviations

BCID: Blood Culture ID Panel
BSI: bloodstream infection
ddPCR: droplet digital polymerase chain reaction
LOD: limit of detection
PCR: polymerase chain reaction
qPCR: quantitative polymerase chain reaction
qSOFA: quick Sepsis-related Organ Failure Assessment
