# Supplementary material for: Multiplex Droplet Digital Polymerase Chain Reaction Assay for Rapid Molecular Detection of Pathogens in Patients With Sepsis: Protocol for an Assay Development Study
Source: JMIR Res Protoc. 2021 Dec 13;10(12):e33746. doi: 10.2196/33746 (PMC8713102; doi:10.2196/33746)
Supplement: Multimedia Appendix 1 [file resprot_v10i12e33746_app1.pdf]

PhD project proposal: SDU-2019-92 - (1617) - Rapid Detection of Pathogens in Blood Culture-Negative Sepsis-
Patients Using Multiplex Droplet Digital PCR
Authors: Ming Chen (Corresponding Applicant) Date submitted: 2019-06-26

Graduate School
Faculty of Health Sciences
SDU

Dear Dr Chen

It is a pleasure to inform you that your project proposal is accepted as basis for enrollment in the PhD programme at the Graduate School of Health Sciences, SDU.
The Graduate School has also approved your student as a potential PhD student.

You as the main supervisor are asked to fill in a form concerning your student's enrolment and the PhD Plan. Please click here to download form:
http://www.sdu.dk/~/media/Files/Forskning/PhD/Phd_sundhedvidenskab/skemavejl/Enrolment_form.doc

Please be aware that in order to enrol your student we must receive documentation that the project is fully financed. This means that you need to have signed documents stating that three years of salary, three years of tuition fees, and at least 50
% of your working/running expenses are guaranteed. This information must be submitted alongside the enrolment form to phd@health.sdu.dk with all relevant signatures.

Please be aware that your student cannot be enroled until the Graduate School has approved the PhD plan (part of the enrolment form). The plan can however change during the course of the PhD programme.
When the Graduate School has approved both the financial plan and the PhD Plan the enrollment procedure will continue.

PhD students are encouraged to make use of the Faculty of Health Sciences' biostatistical consultancy service, when relevant. For more information, please see:
https://www.sdu.dk/en/om_sdu/institutter_centre/ist_sundhedstjenesteforsk/forskning/epidemiologi/vejledning

University of Southern Denmark
Campusvej 55
DK-5230 Odense M
Denmark
T +45 6550 1000
www.sdu.dk

SDU

Sincerely,

Christian Godballe
Head of PhD School
cgodballe@health.sdu.dk
Graduate School - Faculty of Health Sciences, SDU

Internal Reviewer 1 report:
I have read the PhD project 'Rapid Detection of Pathogens in Blood Culture-Negative Sepsis-Patients Using Multiplex Droplet Digital PCR‘ and find it very interesting and important. I do not have any criticisms and recommend the project
----
Internal Reviewer 2 report:
The PhD protocol ‘Rapid Detection of Pathogens in Blood Culture-Negative Sepsis Patients Using Multiplex Droplet Digital PCR’ aim to develop a novel more sensitive molecular method (multiplex ddPCR assay) to detect 4 common bacteria causing sepsis.
The protocol includes 3 substudies. The author will design and develop primers and probes for the PCR assay using S. aureus, S. pneumoniae, E. coli and P. aeruginosa from cultured blood and the highly conserved region of the 16SrRNA gene in the Study 1. The next study is to determine analytical sensitivity and specificity of the assay. The authors will evaluate the new PCR assay in clinical samples from patients admitted with severe sepsis and have negative blood culture on day 5-7.
Sepsis is a life-threatening condition and half of the patients have no positive microbiological results. It is therefore of value to develop rapid and sensitive methods. The study is original and the methods are appropriate. The study plan is realistic, although the study 3 needs to be described better.
There are issues in the protocol that are not quite clear. I suggest the following points to consideration:
The hypothesis does not correlate with the objectives or the methods of the 3 substudies. The correct hypothesis is that the percentage of the culture negative sepsis can be reduced with more sensitive Multiplex Droplet Digital PCR.
Project 3 is a clinical trial and needs to be described better. The feasibility of the sample size in study sites should be discussed.
The supervisor group is well qualified and experts in the research area. The ethical considerations are discussed in detail.
The time frame of 3 years for this project seems reasonable.
With these remarks I recommend this protocol for a relevant and important PhD project.
